# Supplementary material for: Chromatin remodelers couple inchworm motion with twist-defect formation to slide nucleosomal DNA
Source: PLoS Comput Biol. 2018 Nov 5;14(11):e1006512. doi: 10.1371/journal.pcbi.1006512 (PMC6237416; doi:10.1371/journal.pcbi.1006512)
Supplement: S1 Text — (PDF) [file pcbi.1006512.s001.pdf]

Chromatin remodelers couple inchworm motion with twist-defect formation to slide nucleosomal DNA – Supporting Information

Giovanni B. Brandani, Shoji Takada†

Department of Biophysics, Graduate School of Science, Kyoto University, Kyoto 606-8502, Japan

† takada@biophys.kyoto-u.ac.jp

Analysis of nucleosome repositioning

To analyze the sliding of nucleosomal DNA relative to the histone octamer and the remodeler, we use the same approach employed in our previous study on spontaneous nucleosome repositioning [1]. Specifically, we defined a set of continues collective variables that we call contact indexes Δbp_i, one for each of the 14 histone-DNA contact points locates at the half-integer super-helical locations (i= +/-0.5, …, +/-6.5), and one for each lobe of the translocase (i= L1, L2). The contact index Δbp_i evaluates the motion of DNA, in base pairs, at contact point i relative to an initial reference configuration, which is taken to be the 1KX5 crystal structure for the histone contacts and the 5X0Y cryo-EM structure for the translocase contacts. To this aim, we first identify for each contact region the set of DNA phosphate beads and protein Cα beads that are within 10 Å from each other. Then we compute, for each group of beads forming a contact i, a set of root mean square deviations $rmsd_{ij}$ from equally many reference structures, but where the ids of the DNA beads have been shifted to reproduce an ideal screw like motion of DNA by j base pairs (in our analysis j goes from -15 to +15 bp). Let us now consider for instance the histone-DNA contact i. At time 0, the DNA adopts the configuration found in the 1KX5 crystal, so that $rmsd_{i0}$ is close to zero, whereas $rmsd_{ij}$ for j different from zero is significantly larger. On the other hand, if after some time the DNA slides forward by 1 base pair via a rotation-coupled motion at contact point i (as happens in our simulations), then $rmsd_{i1}$ will be now close to zero, whereas all others will take larger values. Then, the number of base pairs by which the DNA moved at a certain contact i relative to the initial configuration is simply given by the index j which gives the smallest rmsd from the reference. We compute this value as a continuous path collective variable Δbp_i as:

$$\Delta bp_i(x) = \frac{\sum_j j \exp(-\lambda\, rmsd_j)}{\sum_j \exp(-\lambda\, rmsd_j)}$$

where λ is a set to 2.3 times the average rmsd between two consecutive references. This expression was originally used to study reaction pathways via molecular dynamics simulation in combination with enhanced sampling methods [2].

Free-energy calculations and Markov state modeling

To characterize nucleosome repositioning induced by the remodeler in the ATP state (Figs 3 and 5 of the main text), we calculated free energy landscapes and kinetics via Markov state modeling (MSM) [4]. To this aim, clustering of the conformations was performed via the Density Peak algorithm [5], computing the local density on the space defined by the contact indexes of the ATPase lobes and the nucleosome at SHLs -1.5, 1.5 and 2.5 (Δbp_{L1}, Δbp_{L2}, $\Delta bp_{-1.5}$, $\Delta bp_{1.5}$ and $\Delta bp_{2.5}$), using a Gaussian kernel of 0.1 bp. These coordinates are

sufficient to describe the slow formation of nucleosome twist defects and translocase motion that controls the repositioning, as DNA motion at other regions is faster [1]. The clustering correctly separates the metastable conformations corresponding to the local free energy minima in the landscapes of Fig 4b and Fig 5b, i.e. states cA0, cA1, cB0, cB1, cC1, cC2, cD1, cD2, o0 and o1. The Markov state models were generated using the software PyEMMA [6] with a lag time of 2.5×10^5 MD steps, after which the estimates of the relaxation time scales of the models remain nearly constant (S3 Fig). For the MSMs of the remodeler (wild type or mutant) on $601_{\Delta 3}$ nucleosomes, the 100 unbiased trajectories were sufficient to obtain a connected set of transitions covering the phase space. For all other systems, due to the higher free energy differences, this approach was not viable, since after the first sliding of nucleosomal DNA induced by the translocase closure we never observe the reverse transitions back to the original configuration, preventing the reconstruction of the full dynamics. To solve this problem, we run for each system 10 additional 10^8-MD-steps simulations with a biasing harmonic potential along the Snf2-DNA rmsd, adjusted so that the initial (cA0, cA1) and final (cD1, cD2) configurations along the remodeling pathways have roughly the same free energy, allowing to observe transitions in both directions. The unbiased free energy landscapes and kinetics can then be reconstructed using the recently-developed transition-based reweighting analysis method (TRAM) for multiensemble Markov models [7]. The free energy profiles reported in Figs 3 and 5 of the main text were generated by reweighting the populations of the MSM clusters along the corresponding collective variables. Errors on the free energy profiles were evaluated by computing the standard deviation of the profiles obtained from distinct sets of trajectories, giving in all cases errors within ~0.3 k_BT. The free energy profiles of spontaneous repositioning in the absence of Snf2 (in Fig 3) were generated by reweighting the conformations observed in 8×10^8-MD-steps simulations, where we introduced a linear bias along the average contact index at SHL 2 to facilitate the uniform exploration of the phase space (especially important for the 601 positioning sequence), and two harmonic walls to prevent sliding by further than 1.5 bp in either direction away from $(\Delta bp_{1.5}+\Delta bp_{2.5})/2=0$. The minimum-energy paths in Fig 5 were determined via the nudged elastic band optimization method [8].

SI References

1. Brandani GB, Niina T, Tan C, Takada S. DNA sliding in nucleosomes via twist defect propagation revealed by molecular simulations. Nucleic Acids Res. Oxford University Press; 2018;46: 2788–2801. doi:10.1093/nar/gky158

2. Branduardi D, Gervasio FL, Parrinello M. From A to B in free energy space. J Chem Phys. 2007;126: 054103. doi:10.1063/1.2432340

3. Luger K, Mäder AW, Richmond RK, Sargent DF, Richmond TJ. Crystal structure of the nucleosome core particle at 2.8 Å resolution. Nature. 1997;389: 251–260. doi:10.1038/38444

4. Prinz J-H, Wu H, Sarich M, Keller B, Senne M, Held M, et al. Markov models of molecular kinetics: Generation and validation. J Chem Phys. 2011;134: 174105. doi:10.1063/1.3565032

5. Rodriguez A, Laio A. Clustering by fast search and find of density peaks. Science. American Association for the Advancement of Science; 2014;344: 1492–6. doi:10.1126/science.1242072

6. Scherer MK, Trendelkamp-Schroer B, Paul F, Pérez-Hernández G, Hoffmann M, Plattner N, et al. PyEMMA 2: A Software Package for Estimation, Validation, and Analysis of Markov Models. J Chem Theory Comput. American Chemical Society; 2015;11: 5525–5542. doi:10.1021/acs.jctc.5b00743

7. Wu H, Paul F, Wehmeyer C, Noé F. Multiensemble Markov models of molecular thermodynamics and kinetics. Proc Natl Acad Sci U S A. National Academy of Sciences; 2016;113: E3221-30. doi:10.1073/pnas.1525092113

8. Sheppard D, Terrell R, Henkelman G. Optimization methods for finding minimum energy paths. J Chem Phys. AIP Publishing; 2008;128: 134106. doi:10.1063/1.2841941
