# Supplementary figures and images for: Chromatin remodelers couple inchworm motion with twist-defect formation to slide nucleosomal DNA
Source: PLoS Comput Biol. 2018 Nov 5;14(11):e1006512. doi: 10.1371/journal.pcbi.1006512 (PMC6237416; doi:10.1371/journal.pcbi.1006512)

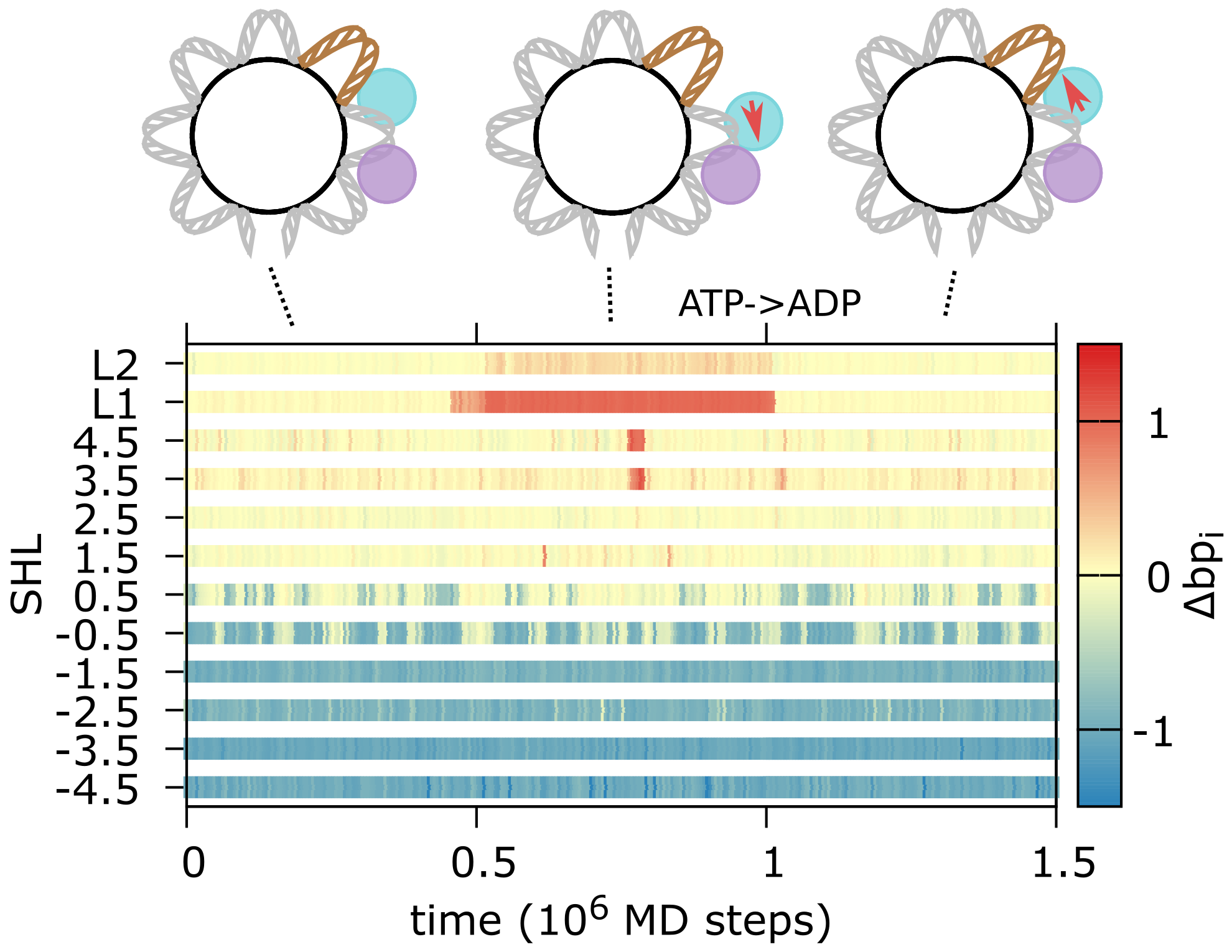

Supplement: S1 Fig — Timeline of the translocase (L1 and L2) and nucleosome contact indexes (SHL -4.5 to 4.5) for a representative trajectory where ATP consumption does not induce nucleosome sliding. ATP binding occurs at time 0, inducing the closure of the remodeler after about 0.5x10^6^ MD steps. Here we induce ATP hydrolysis only after 106 MD steps (instead of 107), so that the remodeler does not have enough time to induce sliding of nucleosomal DNA before the opening of the ATPase domain, which simply causes the system to come back to the initial open conformation because of steric interactions between lobe 2 (in purple) and the nucleosome. (TIF) [file pcbi.1006512.s002.tif]

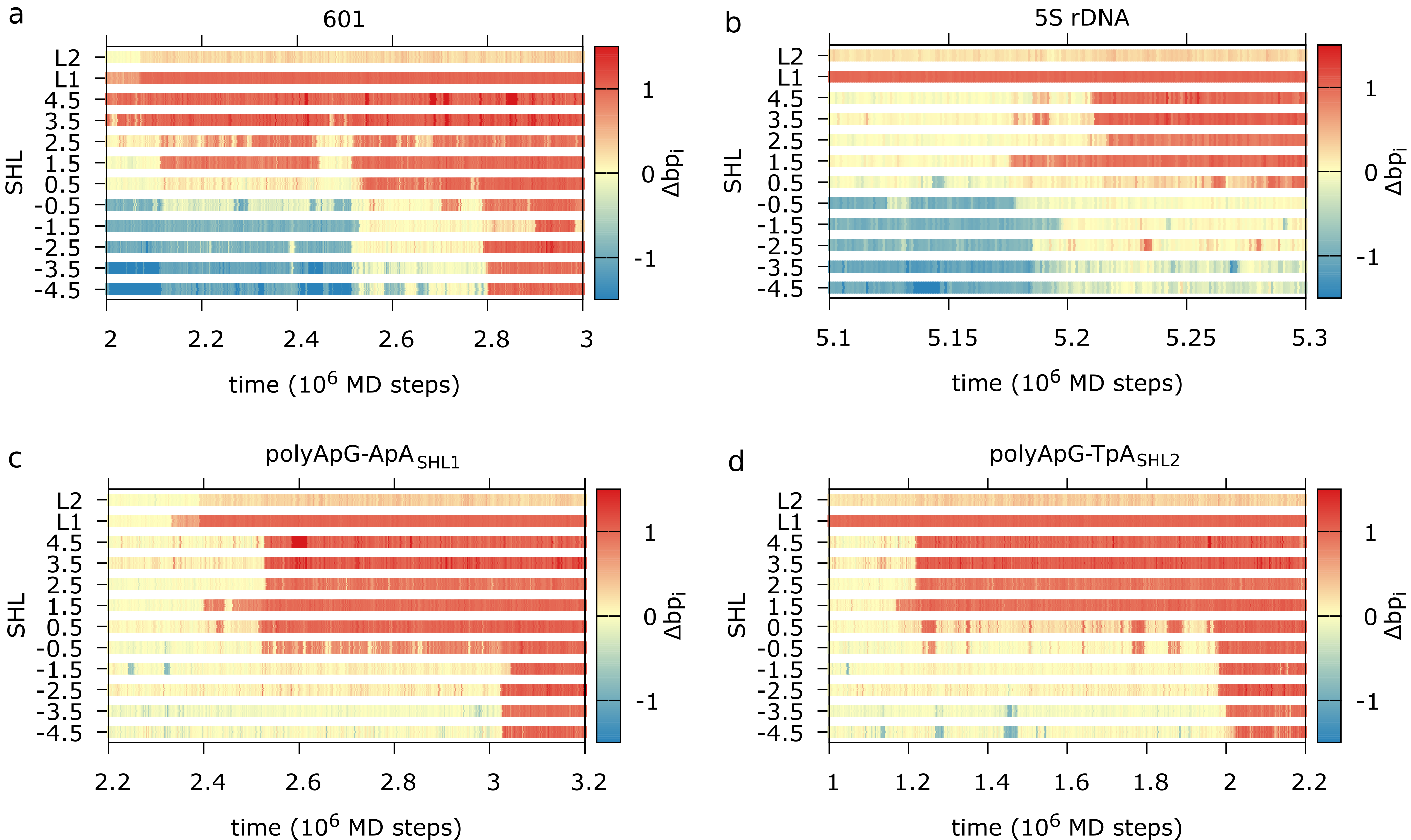

Supplement: S2 Fig — Active nucleosome repositioning via twist-defect propagation on different nucleosomal DNA sequences: (a) 601, (b) 5S rDNA, (c) polyApG-ApASHL1, and (d) polyApG-TpASHL2. We show timelines of the translocase (L1 and L2) and nucleosome contact indexes (SHL -4.5 to 4.5) for representative trajectories where nucleosomal DNA slides by 1 bp relative to the initial configuration. As in Fig 4A from the main text, these plots highlight how nucleosome repositioning occurs via the formation and propagation of twist defects from the remodeler binding location at SHL 2. (TIF) [file pcbi.1006512.s003.tif]

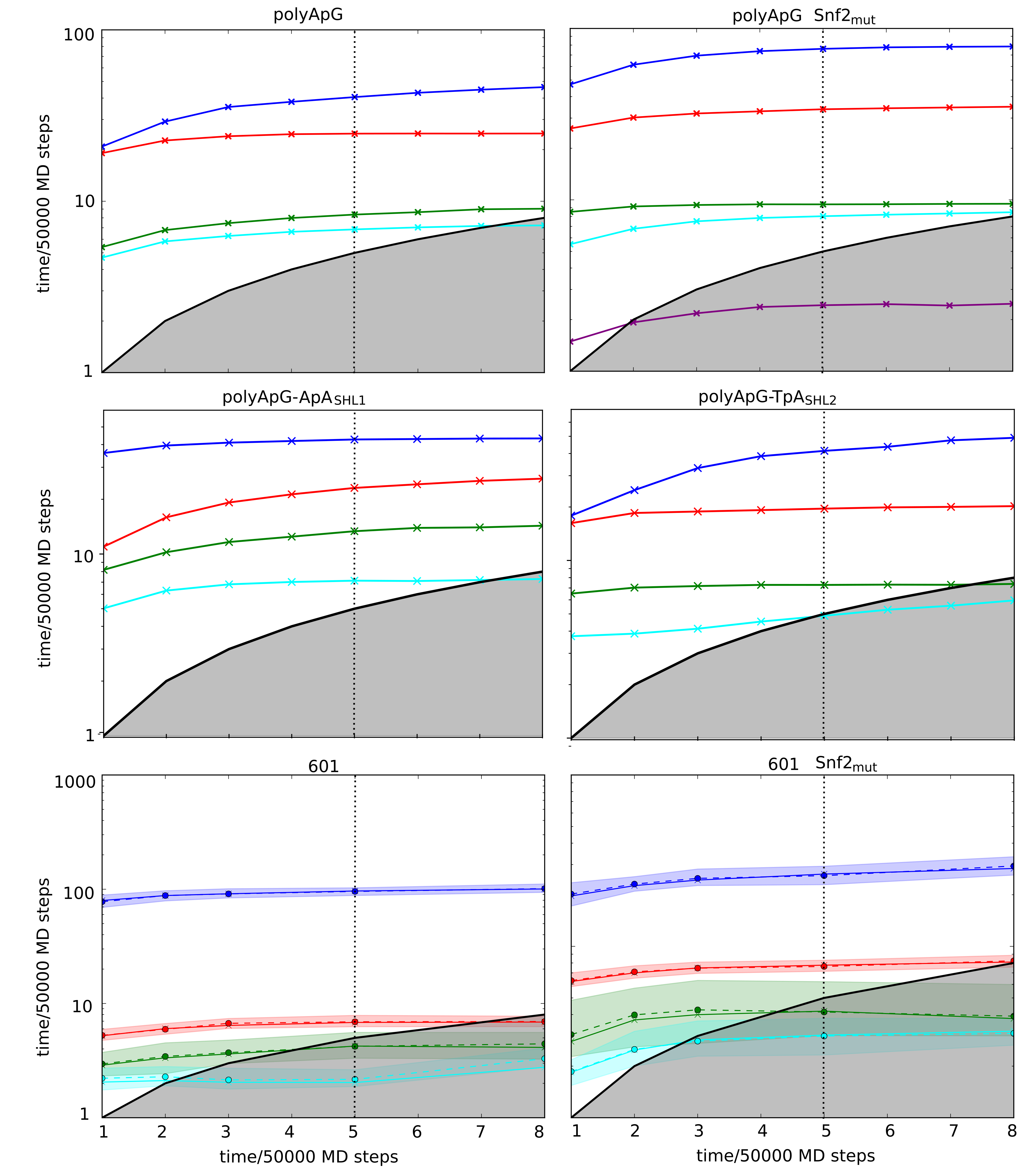

Supplement: S3 Fig — For each considered system, we plot the four slowest relaxation time scales of the MSM as a function of the chosen lag-time. The results reported in the main text were obtained with a lag-time of 2.5x10^5^ MD steps (indicated by the vertical dotted lines), after which the time scales are nearly constant. (TIF) [file pcbi.1006512.s004.tif]

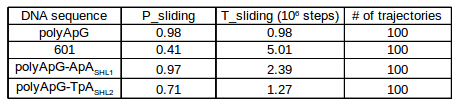

Supplement: S1 Table — (TIF) [file pcbi.1006512.s005.tif]
